# Mouse models of sarcomas: critical tools in our understanding of the pathobiology

**DOI:** 10.1186/2045-3329-2-20

**Published:** 2012-10-04

**Authors:** Sean M Post

**Affiliations:** 1 Department of Leukemia, M.D. Anderson Cancer Center, 1515 Holcombe Blvd, Houston, TX 77030, USA

**Keywords:** Sarcoma, Mouse models, p53, Retinoblastoma (Rb), Translocation

## Abstract

Sarcomas are neoplastic malignancies that typically arise in tissues of mesenchymal origin. The identification of novel molecular mechanisms leading to sarcoma formation and the establishment of new therapies has been hampered by several critical factors. First, this type of cancer is rarely observed in the clinic with fewer than 15,000 newly cases diagnosed each year in the United States. Another complicating factor is that sarcomas are extremely heterogeneous as they arise in a multitude of tissues from many different cell lineages (e.g. bone (osteosarcoma), fat (liposarcoma), and muscle (myosarcoma)). The scarcity of clinical samples coupled with its inherent heterogeneity creates a challenging experimental environment for clinicians and scientists. Faced with these challenges, there has been extremely limited advancement in treatment options available to patients as compared to other cancers. In order to glean insight into the pathobiology of sarcomas, scientists are now using *in vivo* mouse models whose genomes have been specifically tailored to carry gene deletions, gene amplifications, and point mutations commonly observed in human sarcomas. The use of these model organisms has been successful in increasing our knowledge and understanding of how alterations in relevant oncogenic, tumor suppressive, and signaling pathways directly impact sarcomagenesis. It is the goal of many in the biological community that the use of these mouse models will serve as powerful *in vivo* tools to further our understanding of sarcomagenesis and potentially identify new therapeutic strategies.

## Background

Sarcomas are a rare form of cancer with less than 15,000 new cases diagnosed each year in the United States. Though rare, sarcomas are highly debilitating malignancies as they are often associated with significant morbidity and mortality. Sarcomas are biologically very heterogeneous as evidenced by the fact that these cancers arise from a plethora of different tissues and cell types. They are classically defined by their tissue of origin and are additionally stratified by their histopathology or patient’s age at diagnosis [1,2]. While these classifications have proven useful, modern biological and clinical techniques have the ability to further stratify sarcomas based on their genetic profile [1,3,4]. Cytogenetic and karyotype analyses have revealed two divergent genetic profiles in sarcomas. The first and most simple genetic profile is the observation of translocation events in sarcomas with an otherwise normal diploid karyotype. On the other hand, most sarcomas display a more complex genetic phenotype, suggesting genomic instability plays an important role in many sarcomas.

### Historical perspective

Much of our current knowledge regarding sarcoma biology has been ascertained through experimentation using high dose irradiation, viral infections, *in vitro* cell line studies, and xenografts models. One of the earliest animal studies investigated the impact of the Rous sarcoma virus on the development of soft tissue sarcomas [5]. Our knowledge regarding radiation-induced sarcomagenesis largely stems from the observation of women occupationally exposed to radium and animal models subjected to high dose radiation developed sarcomas [6,7]. While the plight of these patients and the subsequent animal experiments led to the identification of a cause and effect for some sarcomas, these observations were unable to identify the molecular events responsible for sarcomagenesis.

To more accurately investigate the genetic and molecular changes manifested in sarcomas, scientists began using patient derived sarcoma cell lines. These cell lines have also added to our understanding of the sarcoma disease progression *in vivo*, through their use in xenograft experiments [8-10]. Even though these cell line experiments have greatly advanced our understanding of sarcomas, they have severe limitations. First, patient-derived cell lines are typically isolated during surgical resection of late stage tumors [11]. Thus, these cells have undergone numerous genetic alterations, complicating our ability to identify the critical primary and secondary genetic causes of these cancers. Second, cell lines isolated from individuals possess diverse genetic backgrounds as humans harbor millions of single nucleotide polymorphic combinations [12]. Finally, some of the currently available sarcoma cell lines have been passaged for more than a generation. The impact of cell culture shock is well documented and no doubt alters the mutation rate and genetic stability of these cell lines [13]. How each of these impacts an individual tumor or its response to therapy is largely unknown.

A second complication arises from the use of immunocompromised xenograft mouse models transplanted with human sarcoma cells. These experiments have the ability to test the tumor forming potential of a particular cell line; however, they fail to faithfully recapitulate the true *in vivo* environment of a sarcoma as they lack a functional immune system [14]. It is widely appreciated that the immune surveillance system plays a critical role in tumor prevention [15]. Furthermore, stromal interactions between the host and the injected cell lines differ significantly and undoubtedly alter normal microenvironment interactions.

Given these caveats, it has become imperative that researchers generate more accurate animal models that will allow scientists to directly investigate the mechanisms of sarcomagenesis. In this review, we will highlight several models engineered to harbor known translocations thought to drive human sarcomagenesis as well as tumor prone models with an increased propensity for sarcoma formation. While this review is not meant to be comprehensive of all sarcoma models, we will discuss how specific genetic alterations, pathways, and animal models may serve as preclinical models for future studies, and thus provide a framework for other studies examining the impact of translocations or deregulated pathways.

### Sarcomas defined by translocation

As alluded to above, some sarcomas harbor diploid karyotypes but posses chromosomal translocation, suggesting a direct correlation between the translocation event and the etiology of the disease [16]. The specificity of individual translocations are likewise useful diagnostic indicators of specific sarcomas. Ewing's sarcomas commonly carry a t(11;22)(q24:q12) reciprocal translocation resulting in a gene fusion product between the RNA binding protein *Ews* and the transcription factor *Fli1*[17,18]. Given that there are fewer than 300 new Ewing’s sarcoma cases in the United States each year, our understanding of the disease process is quite limited. Therefore, in order to directly interrogate the impact of the *EWS-FLI1* fusion gene on tumor formation, several laboratories have generated mouse models expressing an *Ews-Fli1* transgene.

Alveolar rhabdomyosarcomas, like Ewing’s sarcomas, are also often defined by the presence of translocation events, most commonly t(2;13)(q35;q14) and t(1;13)(p36;q14) [19,20]. However, the majority of these are the t(2;13)(q35;q14) translocation which results in the fusion of the transcription factor *Pax3* with the transactivation domain of *Fkhr*[21]. Like Ewing’s sarcoma, alveolar rhabdomyosarcomas are exceedingly rare, with fewer than 100 new cases a year reported in the United States. Since clinical samples are difficult to obtain, our knowledge of this disease is quite sparse. To combat this dilemma, several mouse models mimicking the alveolar rhabdomyosarcoma translocation events have recently been generated. The generation and characterization of the alveolar rhabdomyosarcoma and Ewing’s sarcoma mouse models and their impact on tumor formation will be detailed in later sections.

### Sarcomas with complex karyotypes

In contrast to sarcomas identified as having diploid karyotypes, the majority of sarcomas belong to the more karyotypically complex group. Cytogenetic and karyotypic analyses of undifferentiated pleomorphic sarcomas, pleomorphic rhabdomyosarcomas, embryonal rhabdomyosarcomas, and osteosarcomas have revealed their genomes to be unstable and disorganized as evidenced by multiple deletions, amplifications, and chromosomal fusions [22]. Molecular analyses have shown that many of the canonical tumor suppressor pathways, such as the p53 and retinoblastoma pathways are ablated in these tumors [22]. Furthermore, some sarcomas also harbor activating oncogenic mutations; such as expression of oncogenic K-ras. Together, disruption of these genes and pathways are thought to be a driving force in sarcomagenesis.

Unlike the direct correlation between a single chromosomal translocation event in diploid sarcomas, it is more challenging to identify which of the numerous mutations, deletions, or amplifications drive the development of sarcomas with complex cytogenetics. Furthermore, the extreme heterogeneity in these sarcomas is also a challenge for clinicians attempting to develop personalized treatment strategies. Given these complexities, we will highlight some of the critical pathways thought to be altered during sarcomagenesis below.

### Tumor suppressor and oncogenic pathways involved in sarcomagenesis

#### The p53 pathway

The p53 tumor suppressor pathway is one of the most well characterized pathways in cancers [23]. The *TP53* gene encodes a transcription factor required for the activation of numerous DNA damage-dependent checkpoint response and apoptotic genes [24,25], and thus its activities are often ablated in many cancers. In addition to loss of p53 functions via inherited germline mutations, the p53 pathway is commonly disrupted by point mutations in the *p53* gene during sporadic sarcomagenesis [26]. However, even though *p53* gene alterations are widely regarded as having a significant impact on sarcomagenesis, many sarcomas retain wild type *p53*, yet phenotypically display a loss of p53 function. These findings suggest that changes in other components of the p53 pathway; such as amplification of Mdm2, a negative regulator of the p53 pathway, may result in p53 inactivation [27,28]. Furthermore, both mice and humans with elevated levels of Mdm2 due to a high frequency single nucleotide polymorphism in the Mdm2 promoter (Mdm2SNP309) are more susceptible to sarcoma formation [29-31]. Additionally, deletion or silencing of *p19*^*Arf*^ (*p14*^*Arf*^ in human), an inhibitor of the Mdm2-p53 axis, often results in development of sarcomas. Together, these data indicate that while inactivation of the p53 pathway is observed in the vast majority of human sarcomas, the mechanisms leading to disruption of the pathway can vary greatly.

#### The retinoblastoma pathway

The retinoblastoma (Rb) pathway represents a second major tumor suppressor pathway deregulated in many sarcomas. Individuals inheriting a germline *Rb* mutation typically develop cancers of the eye early in life [32-34]. However, in addition to retinal cancers, these children have a significantly higher propensity to develop sarcomas than the general population [35]. While inheritance of a germline *Rb* alterations increases sarcoma risk, there are also numerous examples of sporadic sarcomas harboring spontaneous *Rb* mutations and deletions, particularly osteosarcomas and rhabdomyosarcomas [36]. Furthermore, *p16*^*Ikn4a*^, a negative regulator of the CDK-cyclin complexes that phosphorylate and activate Rb, is often deleted in sarcomas [37,38]. Together, these findings illustrate the importance of the Rb pathway in sarcomagenesis.

### Oncogenic signaling

In addition to loss of tumor suppressor pathways, sarcomagenesis is also driven by aberrant oncogenic signaling. The Ras signaling pathway in particular is thought to be altered during sarcoma development [39]. Deregulation of the Ras pathway aberrantly stimulates cellular proliferation, which in and of itself impinges on the p53 and Rb pathways, collectively demonstrating the significant cross-talk between these three separate but overlapping pathways.

Given the numerous signaling pathways potentially disrupted in sarcomas, there has been a critical need to interrogate how each of these genes and divergent pathways impact sarcomagenesis in a prospective manner. Since these studies are nearly impossible in human patients, scientists and clinicians are now using mice genetically tailored for such studies (Table 1). Below, we will highlight several well characterized genetically engineered mouse models harboring common genetic alterations observed in sarcoma biology.

**Table 1:** Mouse models of human sarcomas

**Tumor type**	**Gene alteration(s)**	**Agent used**	**Significance**	**Proposed karyotype**	**Reference**
Soft Tissue Sarcoma	ND	Rous sarcoma virus	Viral infection influences sarcomagenesis	Unknown	[5]
Osteosarcoma	ND	Radiation	Radiation influences 7 osteosarcoma formation in rabbits	Unknown	[7]
Sporadic/ Varied	p53−/−	None	Loss of *p53* results in sarcoma formation	Complex	[40,41]
Sporadic/ Varied	*p53*^R172H^ mutation	None	Mutations in p53 results in sarcoma formation	Complex	[42,43]
Sporadic/ 31 Varied	*Mdm2*^SNP309^ w or w/o *p53*^R172H^mutation	None	Mutations in the p53 pathway result in sarcoma formation	Complex	[31]
Poorly Differentiated Sarcoma	*Ews-Fli1*^Tg^ w or w/o *p53 *deletion	*Prx-Cre*	*Ews-Fli1*^Tg^mice fail to induce sarcomagenesis in the absence of *p53 *loss	Translocation but complex with p53/Rb loss	[44]
Poorly Differentiated Sarcoma/ Osteosarcoma	Ews-Fli1^Tg^ w or w/o Rb and p53 deletions	*Prx-Cre*	*Ews-Fli1*^Tg^*;Rb*^−/−^ mice fail to induce sarcomagenesis but reduce time of onset in the absence of *p53*	Translocation but complex with *p53/Rb *loss	[45]
Osteosarcoma	*Tax*^Tg^*;p19Arf*^−/−^	None	Expression of Tax in the absence of *p19Arf* results in osteosarcoma formation	Complex	[46]
Osteosarcoma	*Rb and p53* deletions	*Osterix*-Cre	Deletion of *p53* and *Rb* cooperate in the bone leading to osteosarcomagenesis	Complex	[47]
Osteosarcoma/ Leiomyosarcoma	Rb ^+/-^ and p107 deletions	None	*Rb* haploinsufficiency coupled with *p107* deletion results in low penetrant Sarcoma formation	Complex	[48]
Developmental defects	*Pax-3-Fkhr* fusion	None	Pax3-Fkhr fusion product fails to produce sarcomas when expressed from Pax3 promoter	Translocation	[49]
Rhabdomyosarcomas	*Pax3-Fkhr* transgene w/ and w/o *Rb* and *p53* deletion	*Myf6-Cre*	Expression of the Pax3-Fkhr transgene requires loss of *p53* and *Rb* for rhabdomyosarcoma formation	Translocation but complex with *p53/Rb* loss	[50,51]
Rhabdomyosarcomas/ Various sarcomas	*Ptch1 heterozygosity* w/ and w/o *Rb* and *p53* deletion	*Myf6-Cre, Myf-5Cre,* or *Pax7-CreER*	Loss of *p53* and *Rb* in conjunction with *Ptch1* haploinsufficiency results in rhabdomyosarcomagenesis	Complex	[52]
Undifferentiated Pleomorphic sarcomas	Mutant K-ras expression and *p53* loss	Adenoviral Cre	MutantK-ras expression and *p53* loss cooperate in the development of undifferentiated pleomorphic sarcomas	Complex	[53,54]
Pleomorphic Rhabdomyosarcomas	Mutant K-ras expression and *p53* loss	Electroporation of Cre into the muscle	MutantK-ras expression and *p53* loss cooperate in the development of undifferentiated pleomorphic sarcomas	Complex	[55]
Rhabdomyosarcomas	Mutant K-ras expression and *p53* loss or mutation of *p53*	*Ah-Cre*	Expression of mutant p53 facilitates a more rapid development of rhabdomyosarcomas than loss of *p53* in the mutant K-ras background	Complex	[56]

### Mouse models of sarcomas

For many years, mouse models have served as powerful tools in our interrogation of the mechanisms regulating human cancers. However, it was not until the prevalence of genetically manipulable mouse models in the 1980’s and 90’s that we became fully capable of examining the direct causes of many cancers in an *in vivo* setting. Although we do not fully understand the disease processes of sarcomagenesis, we now have ample biological reagents in which to explore these processes, several of which are detailed below.

### Mouse models harboring translocations

#### Ewing’s Sarcoma

Sarcomas with simple diploid karyotypes often have chromosomal translocations that directly impact sarcomagenesis. To identify the impact of the *Ews-Fli1* translocation, t(11;22)(q24:q12), in Ewing’s sarcoma, mice harboring an *Ews-Fli1* transgene have been generated. Expression of the *Ews-Fli1* transgene is lethal when expressed in some tissues [57]. Therefore, to limit this lethal phenotype, the *Ews-Fli1* transgene must be conditionally expressed in specific cell types using the Cre-recombinase-loxP system [58]. Cre-loxP technologies have the ability to delete entire genes, specific exons, or even remove inhibitors of transgenic expression in specific cell lineages or tissues [59]. Using this system, transgenic mice harboring a latent *Ews-Fli1* transgene were generated and crossed with mice expressing Cre-recombinase under the control of the *Prx*-promoter [44], resulting in the activation of the *Ews-Fli1* transgene specifically in osteogenic multipotent cells. Although these *Prx-Cre;Ews-Fli1* mice developed multiple bone abnormalities, they ultimately failed to produce sarcomas. This finding suggests that while the t(11;22)(q24:q12) translocation is a common event in Ewing’s sarcoma, it is, by itself, unable to stimulate a cancer phenotype which indicates that other accompanying mutations (or “hits” to the genome) are required for frank tumor formation. To address this, mice expressing the *Ews-Fli1* transgene were then crossed to mice harboring Prx-Cre-directed deletion of *p53*. The *Prx-Cre;Ews-Fli1;p53*^*−/−*^ mice rapidly developed poorly differentiated sarcomas (median age of 21 weeks); while *Prx-Cre* mediated deletion of *p53* alone resulted in the development of osteosarcomas (median age of 50 weeks), demonstrating the cooperative interactions between Ews-Fli1 and p53 in sarcomas.

### Alveolar rhabdomyosarcomas

Alveolar rhabdomyosarcomas are often characterized by t(2;13)(q35;q14) translocations. Knock-in mice harboring the t(2;13)(q35;q14) translocation have been generated by knocking the *Fkhr* gene into the *Pax-3* locus, resulting in a *Pax-3-Fkhr* fusion gene under the control of the endogenous *Pax-3* promoter [49]. Similar to the *Prx-Cre;Ews-Fli1* studies, these mice did not develop sarcomas, but did display numerous congenital defects, suggesting the *Pax3-Fkhr* fusion gene is important in normal murine development but requires additional genetic hits for sarcoma development. In order to generate a more robust alveolar rhabdomyosarcoma model, mice specifically expressing a *Pax3-Fkhr* transgene in the muscle under the influence of *Myf6-Cre*-mediated activation were generated [50,51]. Surprisingly, these mice also failed to display a sarcoma phenotype. However, concomitant deletion of *p53*, *p19*^*Arf*^, or *p16*^*Ink4a*^ in the *Myf6-Cre;Pax3-Fkhr* mice resulted in a rhabdomyosarcoma phenotype [50,51]. These studies illustrate the complexities in alveolar rhabdomyosacromagenesis and implicate the p53 and Rb pathways in the development of Pax3-Fkhr-dependent sarcomas.

## Additional sarcoma mouse models regulated by transloaction events

### Synovial sarcomas/myxoid liposarcomas

The identification of common translocation events has greatly assisted in our understanding of sarcomagenesis and has led to the generation of mouse models with the power to examine their impact. In addition to the translocations noted above, chromosomal rearrangements t(X;18) and t(12;16) (q12;p11) are commonly observed in synovial and liposarcomas, respectively (Table 2). Mouse models mimicking the t(X;18) translocation, via expression of the chimeric protein SYT-SSX2, result in synovial sarcomas with high penetrance [60,61]. Likewise, expression of TLS-CHOP, a fusion protein that mimics the t(12;16) (q12;p11) translocation, resulted in myxoid round cell liposarcomas [62]. Given the rare nature of these tumors, these mouse models make excellent platforms for investigating the pathobiology of these diseases as well as pre-clinical therapeutic models [76,77].

**Table 2:** Additional mouse models of human sarcomas

**Tumor type**	**Gene alteration(s)**	**Agent used**	**Significance**	**Proposed karyotype**	**Reference**
Synovial Sarcoma	SYT-SSX2 fusion	*Myf 5-Cre*	Expression of the SYT-SSX2 transgene resulted in 100% penetrant synovial sarcomas	Translocation	[60,61]
Myxoid Liposarcoma	TLS-CHOP fusion w/ p53 deletion	*Prx-Cre*	Deletion of *p53 *cooperates in the formation of liposarcomas	Translocation	[62]
Neurofibroma MPNST	*NF1 *deletion w or w/o *p53 *and *p19Arf *deletions	Germline, *POa-Cre*, or *3.9Periostin-Cre*	Deletion of *p53 *and p19Arf stimulate MPNST development	Complex	[63,64]
Uterine leiomyosarcoma	*Lmp2 deletion*	None	Loss of Lmp2 results in uterine leiomyosarcoma formation	Complex	[65,66]
Uterine leiomyosarcoma	TDGF1/CRIPTO overexpression	MMTV-promoter	TDGF1/CRIPTO expression results in uterine leiomyosarcoma development	Complex	[67]

## Sarcoma mouse models with complex genetics

### Sarcomas of the bone (osteosarcomas)

In contrast to the sarcomas driven primarily by specific translocations, the majority of sarcomas possess highly aneuploid genomes due to disruptions in tumor suppressor pathways and aberrant oncogenic activation. Osteosarcomas are one of the most well studied types of sarcomas with complex genetics given the development of numerous knock-out, knock-in, and transgenic animal models available for this disease. The generation and characterization of tumors from *p53*-null and *p53*-heterozygous knock-out mice demonstrated the importance of p53 in osteosarcomas [40,41]. The role of p53 in osteosarcomas is further highlighted by tumor analysis of *p53* knock-in mice containing a mutant copy of p53R172H (corresponding to the R175H hot-spot mutation in humans) [42,43]. An important differentiation between the *p53* knock-out and *p53*^*R172H*^ knock-in mice is that *p53*^*R172H*^ sarcomas developed a metastatic gain of function phenotype, faithfully recapitulating the phenotype observed in the human disease [42,43]. The generation of the mutant *p53*^*R172H*^ mouse model provides researchers, for the first time, with the ability to investigate metastatic osteosarcoma disease progression in a truly *in vivo* setting. In addition to direct ablation of p53 function, transgenic mice overexpressing the p53 regulator, Mdm2, as well as mice harboring a single nucleotide polymorphism in the Mdm2 promoter, have an increased risk to develop sarcomas [31,68]. Furthermore, transgenic mice expressing the viral oncogene *Tax*, coupled with deletion of *p19*^*Arf*^, developed highly penetrant osteosarcomas [46]. Together, these results further demonstrate the importance of ablating the p53 pathway in osteosarcomagenesis.

In humans, loss of the Rb pathway has also been implicated in the etiology of osteosarcomas. However, in the mouse, homozygous deletion of *Rb* results in an embryo lethal phenotype due to placental defects [69]. Therefore, in order to investigate the role of Rb in bone malignancies, researchers again employed the Cre-loxP system to delete *Rb* specifically in the bone. Unlike the critical role of Rb in human osteosarcomas, mice lacking *Rb* in osteocytes do not develop cancers [47]. However, when coupled with *p53* loss, *Rb* loss exacerbates the p53-dependent osteosarcoma phenotype, with most mice succumbing to their disease within 150 days [45,47]. As a caveat to the finding that *Rb*-loss alone did not induce osteosarcomas, there is significant redundancy in the Rb pathway in mice. Rb consists of three family members (p105, p107, and p130) and each shares similar structure and function [70]. As such, concomitant loss of both *Rb* and *p107* in mouse did in fact result in a low penetrant osteosarcoma phenotype [48,71]. Taken together, these studies demonstrate the absolute requirement for ablation of the p53 pathway in osteosarcomagenesis and suggest that pRb plays a co-operative role in osteosarcomagenesis.

## Soft tissue sarcomas

### Undifferentiated pleomorphic sarcomas

Undifferentiated pleomorphic sarcomas are soft tissue sarcomas typically observed in adults that arise from cells of unknown origin, and, like osteosarcomas, display complex genetics resulting from deregulation of multiple pathways. Investigations into the cellular origin of both undifferentiated pleomorphic sarcomas and embryonal rhabdomyosarcomas have identified the importance of the p53 and Rb pathways in the etiology of both malignancies [52]. In addition to the importance of these two tumor suppressor pathways, the Kras-signaling pathway has also been implicated in the development of undifferentiated pleomorphic sarcomas [53,54]. Mice harboring a latent copy of oncogenic *Kras*^*LSLG12D*^ (silenced by a floxed “loxP-stop-loxP” (LSL) cassette) and two floxed *p53* alleles (*p53*^*FlΔ2-10*^) that were simultaneously activated to express mutant *Kras*^*G12D*^ and delete *p53* following injection of adenoviral-Cre into the muscle, rapidly developed sarcomas with significant metastatic potential. Detailed molecular analysis of the *Ad-cre;Kras*^*G12D*^*;p53*^*−/−*^ tumors revealed an expression profile similar to those observed in human undifferentiated pleomorphic sarcomas [54]. Together, these data support the idea that both ablation of tumor suppressor pathways and activation of oncogenes cooperate to drive sarcomagenesis.

### Rhabdomyosarcomas

Using the *Cre-LoxP* strategy to simultaneously activate a latent oncogenic *K-ras*^*G12V*^ allele and delete the *p53*^*FlΔ2-10*^ alleles in myocytes, it was demonstrated that mice rapidly develop sarcomas that are histopathologically similar to pleomorphic rhabdomyosarcomas observed in humans [55]. Although the undifferentiated pleomorphic and rhabdomyosarcoma studies used similar mouse models to identify the role of mutant K-ras and *p53*-loss in sarcomagenesis, these experiments resulted in somewhat different malignancies. Thus, given the cellular similarities between undifferentiated pleomorphic sarcomas and rhabdomyosarcomas [52], it is imperative to further investigate sarcomagenesis in the *Kras-*^*LSLG12D*^*;p53*^*Fl2Δ10/Fl2Δ10*^ mouse models using multiple myospecific Cre-expressing transgenic mice in order to precisely ascertain how these pathways synergies in specific tissues.

While each of the *Kras-*^*LSL*^*;p53*^*Fl2Δ10/Fl2Δ10*^ studies mentioned above reveal the importance of p53 and K-ras in myocyte specific sarcomagenesis, they failed to accurately represent the most common type of alteration to the *p53* gene in human cancers (e.g. p53 mutations). A recent study examined the impact of p53 in sarcomagenesis more accurately by not only deleting *p53* but also expressing the *p53*^*R172H*^ mutant (corresponding to the human p53R175 hotspot mutation) in the muscle [56]. Using the *Kras*^*LSLG12V*^*;p53*^*Fl2Δ10/Fl2Δ10*^ and *Kras*^*LSLG12V*^*;p53*^*R172H/Fl2Δ10*^ alleles in combination with Ah-Cre expression, it was revealed that expression of mutant p53, even in the context of heterozygosity (e.g., p53R172H/+), had a more deleterious effect than simply losing one wild type *p53* allele. These *Ah-Cre;Kras*^*G12V*^*;p53*^*R172H/−*^ mice formed rhabdomyosarcomas with high penetrance as compared to less than 10 % rhabdomyosarcomas formation in the *Ah-Cre;Kras*^*G12V*^*;p53*^*+/−*^ mice. In addition, unlike the tumors from *Ah-Cre;Kras*^*G12V*^*;p53*^*−/−*^ mice, the tumors from the *Ah-Cre;Kras*^*G12V*^*;p53*^*R172H/−*^ mice also recapitulated the metastatic phenotype typically observed in human rhabdomyosarcomas.

## Additional sarcoma mouse models regulated by driver mutations

### Neurofibromatosis/leiomyosarcomas

Given the extreme heterogeneity of sarcomas with regards to tissue of origin, it is obvious that alterations to numerous genes, pathways, and signaling complexes play an important role in the pathobiology of sarcomas. While this review does not cover all genetic alterations responsible for sarcoma development, there are numerous additional genes that impact this disease (Table 2). For example, alterations in expression of tumor suppressor genes, such as Neurofibromatosis type 1 (NF1), likewise impact the etiology of some sarcomas. Mouse models that carry genomic deletions and/or tissue-specific Cre-mediated deletion of *NF1* result in neurofibromas [72]. These NF1-dependent phenotypes are further exacerbated when *NF1* is concomitantly deleted with other tumor suppressors (e.g.; *p53* and *p19*^*ARF*^) resulting in more aggressive phenotypes as evidenced by malignant peripheral nerve sheath tumor formation [63,64]. To further illustrate that loss of a single gene impacts sarcoma formation, mice harboring an *LMP-2* deletion resulted in spontaneous uterine leiomyosarcomas [65]. This provides evidence of its role as a tumor suppressor and a potential biomarker in human disease [66,73]. In addition to loss of function alterations, overexpression of teratocarcinoma-derived growth factor 1, also known as CRIPTO, results in leiomyosarcomas by deregulation of the WNT pathway [67].

## Conclusion

The vast differences in the cellular origins of sarcomas, the lack of availability of tumor specimens, and the heterogeneity inherent within individual tumors has impeded our ability to fully understand the biology of sarcomas. However, given the availability of numerous genetic knock-outs, knock-ins, and conditional alleles coupled with the bevy of tissue-specific Cre-recombinase expressing mouse lines, we now have the ability to systematically and prospectively interrogate how individual genes and mutations impact sarcomagenesis. Going forward, tumor analysis from multiple murine derived tumor types can be compared and contrasted in order to identify critical changes in specific sarcomas. These mouse models have clearly demonstrated that while there are driver mutations/translocations, sarcomagenesis is, in fact, a multi-hit disease. The use of these mouse models mimicking the human disease condition leads to a critical question: what therapeutic approaches can be taken to lessen the impact of these debilitating diseases? First, we must recognize that these mouse models demonstrate the synergism between multiple pathways and thus combinatorial treatment strategies are needed to combat these cancers. For treatment of patients with translocations, one can envision a targeted therapeutic approach, like that which has been observed in the treatment of chronic myeloid leukemia. The addition of tyrosine kinase inhibitors (TKIs), such as imatinib, which inhibits the activity of the BCR-ABL fusion gene, has reduced CML from a death sentence to a manageable and stable disease. Can the scientific/clinical community design target drugs to the translocation events observed in sarcomas? The use of these mouse models may serve as an excellent preclinical platform for such studies.

Treating and alleviating the disease process in sarcomas with complex genetics may prove more difficult than identifying targeted therapies. However, given that many groups have identified the importance of specific pathways in sarcomagenesis, such as the p53 pathway, we have a starting point. Preclinical drugs like PRIMA1-Met and NCS319726 have been shown to restore mutant p53 activities [74,75]. These drugs could be rapidly screened for efficacy in mutant *p53* sarcoma models. Moreover, the p53 pathway is also inactivated by dysregulation of its protein partners, Mdm2 and p19^Arf^. The employment of Mdm2-p53 antagonists, such as Nutlin-3 and RITA may prove efficacious in reactivating the p53 pathway and thus provide a therapeutic benefit. Also, loss of p19^ARF^ due to promoter methylation is a common event in sarcomagenesis. Therefore, these animal models may prove useful in examining the impact of hypomethylating agents, such as azacytidine or dasatinib, in sarcomas.

In cases where specific oncogenes are known to drive tumor formation, such as activated K-ras, the use of compounds that inhibit K-ras targets (such as MEK) could be beneficial. The efficacy of a MEK-inhibitor like ARRY-162 could be readily examined in mouse models possessing a mutated K-ras signaling pathway. All of these potential chemotherapeutic agents, if proven effective in *in vivo* preclinical models, could provide a rationale for personalized and targeted therapy in sarcoma patients.

While mouse models can not completely predict the outcome of each disease, they can provide valuable and critical information, particularly in exceedingly rare types of sarcomas or when low penetrant single nucleotide polymorphisms confound data analysis.

## Abbreviations

Rb: Retinoblastoma; Cre: Cre-recombinase.

## Competing interests

The author declare that he have no competing interests.

## Authors’ contributions

SMP wrote the manuscript.
